# Effect of microstructure on the impact toughness and temper embrittlement of SA508Gr.4N steel for advanced pressure vessel materials

**DOI:** 10.1038/s41598-017-18434-3

**Published:** 2018-01-09

**Authors:** Zhiqiang Yang, Zhengdong Liu, Xikou He, Shibin Qiao, Changsheng Xie

**Affiliations:** Institute for Special Steels, Central Iron and Steel Research Institute Group, Beijing, 100081 China

## Abstract

The effect of microstructure on the impact toughness and the temper embrittlement of a SA508Gr.4N steel was investigated. Martensitic and bainitic structures formed in this material were examined via scanning electron microscopy, electron backscatter diffraction, transmission electron microscopy, and Auger electron spectroscopy (AES) analysis. The martensitic structure had a positive effect on both the strength and toughness. Compared with the bainitic structure, this structure consisted of smaller blocks and more high-angle grain boundaries (HAGBs). Changes in the ultimate tensile strength and toughness of the martensitic structure were attributed to an increase in the crack propagation path. This increase resulted from an increased number of HAGBs and refinement of the sub-structure (block). The AES results revealed that sulfur segregation is higher in the martensitic structure than in the bainitic structure. Therefore, the martensitic structure is more susceptible to temper embrittlement than the bainitic structure.

## Introduction

Reactor pressure vessels (RPV) are generally considered to be the most critical components in the nuclear plant beside the reactor core^1,2^. The RPV should serve for 40 years under high-temperature, high-pressure, and neutron-irradiation conditions. Therefore, to ensure safe operation of the reactor, the RPV should be fabricated from material with high strength, high fracture toughness, and high resistance to irradiation embrittlement^3–5^.

The RPV is typically fabricated from ASME SA508 Class 3 steel^6–8^. With the development of generation IV reactors, larger and higher-power nuclear power plants (compared with those housing generation III reactors) are being built. However, the low strength (620 MPa) of the SA508 Class 3 steel renders this material unsuitable for generation IV reactors and, hence, new materials must be developed. Many countries have considered SA508Gr.4N steel as a candidate material^9,10^.

The RPVs are large forgings and with wall thickness of more than 200 mm^11^. Generally, the forged RPVs are quenched and tempered, as result, the through-thickness microstructure vary significantly because of the differential cooling rates during quenching. These differing microstructures will lead to heterogeneous mechanical properties along the thickness direction of the RPV. Therefore, an understanding of the microstructure and mechanical properties of RPV steels with different quenched microstructures is essential.

The microstructure of the initial quenched martensite is a key factor in determining the performance of RPVs. Park *et al*.^12^ reported that the strength of a quenched Ni–Cr–Mo low alloy steel with a fully martensitic microstructure is significantly higher than that of the quenched microstructure composed of bainite. Similarly, compared with bainite, tempered martensite is more susceptible to temper embrittlement. Raoul *et al*.^13^ found that, in high-P (0.017%) A533 steel, P segregation in martensite is considerably higher than that in bainite. Furthermore, Lee *et al*.^14^ determined the effect of martensitic fraction on the cleavage fracture toughness of a Ni–Cr–Mo steel. The results revealed that the fraction of martensite increases with increasing quenching cooling rate, thereby resulting in a decrease in the fraction of large carbides. Marini *et al*.^15^ investigated the effect of bainitic and martensitic microstructures on the embrittlement of an A508 steel subjected to neutron irradiation. The results revealed that, compared with the fully bainitic microstructures, the martensitic microstructures are more susceptible to non-hardening embrittlement. Gurovich *et al*.^16^ have reported that the critical brittleness temperature of the initial state of a VVER-100 RPV steel increases with the grain size. Moreover, the concentration of P on the grain boundaries increases with increasing duration of the embrittlement heat treatment and, in turn, will accelerate temper embrittlement. Although these studies have elucidated the influence of the initial microstructure on the mechanical properties of Ni–Cr–Mo steels, the relationship among the microstructure, impact toughness, and temper embrittlement of SA508Gr.4N steels remains unclear.

In this work, the microstructure associated with different states of a SA508Gr.4N steel is examined via scanning electron microscopy (SEM), electron backscatter diffraction (EBSD), transmission electron microscopy (TEM), and Auger electron spectroscopy (AES). The mechanical properties are evaluated through tensile tests at room temperature and Charpy impact tests performed at temperatures ranging from −192 °C to 100 °C. The effect of impact toughness and temper embrittlement are discussed from the viewpoint of carbide size, block size, fraction of high-angle grain boundaries (HAGBs) and grain boundary characteristics.

## Materials and Methods

### Material and heat treatments

The chemical composition of the test steel (SA508 Gr.4N) with elemental composition as per ASME boiler and pressure vessel-section – materials–SA-508/SA-508M specification^17^. The chemical composition of the steels is given in Table 1. The carbon content was determined from the GB/T223.69-2008 steel and alloy carbon content. After combustion in the tube furnace, the gas volumetric method was adopted. The concentrations of Al, Ni, Cr, Mo, Mn, V, Ti, Nb were used to determine the multi-element content of GB/T223.79-2007 iron and steel via X-ray fluorescence spectrometry. In the testing standard, the minimum detectable content of P, S, Ti is 0.001%, and the lowest detectable content of Si, Mn, Cu, Al, Nb, Cr, Mo, V is 0.002%.

**Table 1:** Chemical composition of the SA508 Gr.4N steel (wt%).

Material	Measured composition						
	C	Ni	Cr	Mo	Mn	Si	S
ASME SA-508/SA-508M	≤0.23	2.80–3.90	1.50–2.00	0.40–0.60	0.20–0.40	<0.40	<0.02
Test steel	0.19	3.73	1.69	0.50	0.28	0.15	0.003
**Material**	**Measured composition**						
	**P**	**Al**	**Cu**	**V**	**Nb**	**Ti**	**Fe**
ASME SA-508/SA-508M	<0.02	<0.025	<0.25	<0.03	<0.01	<0.015	Bal.
Test steel	0.002	0.002	0.004	0.003	0.005	0.001	Bal.

The steel was melt in vacuum induction furnace, with vacuum pouring in a 100 kg mold, and forged into square and round bars, of 14 × 14 mm and 16 mm diameter respectively, between 1150 °C and 900 °C.

**Figure 1:**
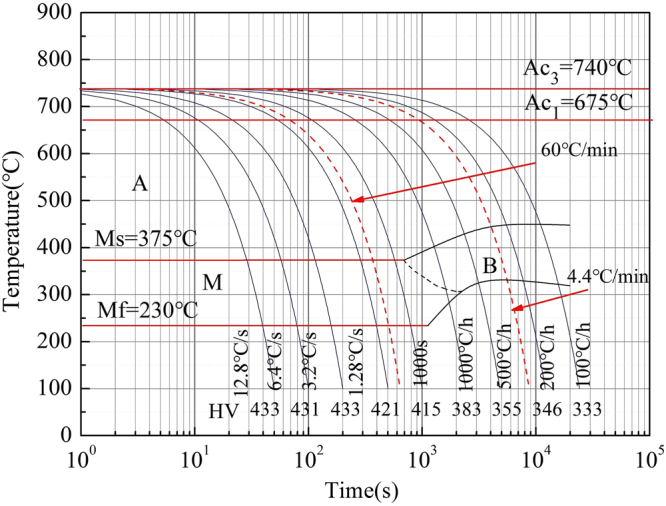
CCT diagram for the SA508Gr.4N steel austenitized at 860 °C.

**Figure 2:**
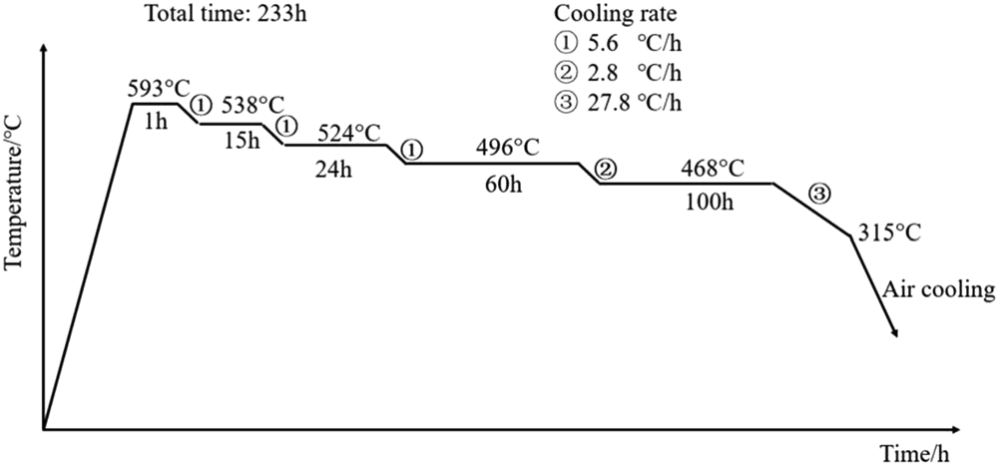
Step-cooling heat treatment.

The steel was subjected to a heat-treatment process (see Table 2) consisting of heating at 920, 900, and 640 °C for 2, 2, and 8 h, respectively. Subsequently, the steel was austenitized at 860 °C for 5 h. To obtain different quenched structures, the quenching cooling rate (QCR) was determined from the continuous cooling transformation (CCT) curve. The critical phase transformation point and CCT curve of the steels were measured with a thermal dilatometer (Fuji Electronic Industrial, Formastor–F) according to the Chinese standard YB/T 5127–1993 and YB/T 5128–93. The specimens cut from the steel bars were machined into long *Φ* 3 × 10 mm cylinders. These cylinders were then heated to 860 °C at a rate of 10 °C/s, austenitized for 5 min at this temperature, and then cooled to room temperature at linear cooling rates of 12.8, 6.4, 3.2, 1.28, 0.64, 0.28, 0.14, 0.07, 0.03 °C/s. The CCT curve was drawn based on the experimental data and is shown in Fig. 1. The CCT curves show that a critical cooling rate (*V*_c_) of ∼35 °C/min is required for martensite formation. Therefore, QCRs of 60 °C/min (martensite) and 4.4 °C/min (bainite) were applied (surface and 1/4 *T*, Wall thickness: *T* = 700 mm). Afterward, the steel was tempered at 620 °C for 8 h. All the specimens were then removed from the furnace and exposed to air until they were cooled to room temperature. Part of the sample was removed to determine the mechanical properties of the material from tensile and impact tests. Another part of the sample was subjected to a step-cooling heat treatment (see Fig. 2) that promotes temper-embrittlement. Then, the performance and microstructure analyses were carried out.

**Table 2:** Heat Treatment process of SA508Gr.4N Steel.

Heat treatment	Pre-heat-treatment	Austenitizing	Tempering
Condition	920/2/AC	860/5/60, 4.4	620/8/AC
[°C/h/C.R (cooling rate)	900/2/AC		
(AC, air cooling. °C/min)]	640/8/AC

### Tensile and Charpy impact tests

The mechanical properties were evaluated through uniaxial tensile testing (NCS GNT300) of ASTM-E8 standard specimens (diameter: 5 mm, gauge: 25 mm) at room temperature. Impact transition curves were obtained from Charpy impact testing of full-size (10 mm × 10 mm × 55 mm) specimens with a 2 mm-long 45° V-notch. The V-type impact test was carried out with a square having dimensions of 14 × 14 mm processing. The notched direction was perpendicular to the square bar. Testing was performed at temperatures ranging from −192 °C to 100 °C, on an NI-500 impact testing machine with a maximum capacity of 500 J.

### Microstructural analysis

The specimens for optical microscopy (OM, Zeiss 40MAT) and SEM (Hitachi S4300) were polished and etched with a 4% nitric acid +96% ethanol solution. Further details of the microstructure were revealed via EBSD (FEI Quanta 650 with Nordlys Nano) and TEM (Hitachi H800). Specimens for EBSD and TEM were prepared through conventional dual-jet electro-polishing in the solution of 8% perchloric acid +92% ethanol at a temperature of −25 °C. In addition, grain boundary segregation of elements after the step-cooling heat treatment was investigated via AES (Ulvac-PHI-700).

## Results

### Microstructure of the SA508Gr.4N steel in the quenched state

**Figure 3:**
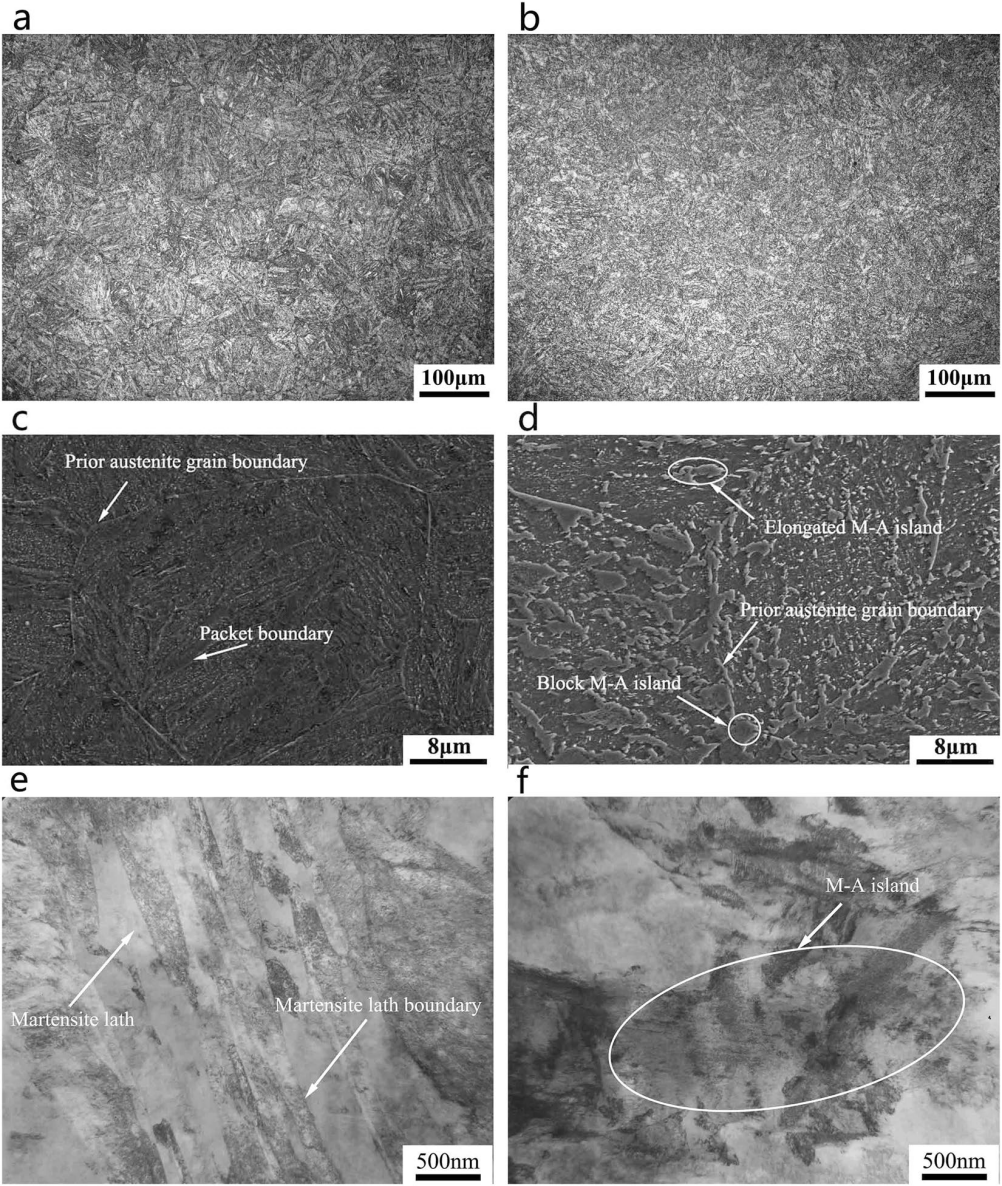
Optical, SEM, and TEM micrographs of the SA508Gr.4N steel quenched at rates of: (**a**,**c**,**e**) 60 °C/min (martensite); (**b**,**d**,**f**) 4.4 °C/min (bainite).

Figure 3 shows the micrographs of SA508Gr.4N steels in the as-quenched state. A quenching rate of 60 °C/min yields an almost lath martensite microstructure (Fig. 3a) with lath widths ranging from 60 nm to 340 nm (Fig. 3e).

Packets with different extension directions occur within the prior austenite grains, and the blocks within the same packets were approximately parallel (Fig. 3c). A quenching rate of 4.4 °C/min yields a granular bainite structure composed of a bainitie ferrite matrix and islands of martensite and austenite (M-A island; see Fig. 3d,f). This microstructure consists of two types of M-A islands (see Fig. 3d), namely: elongated islands distributed in the bainitic ferrite matrix, and blocky islands located primarily at the interface between the matrix and the prior austenite grain boundaries. The martensite within the M-A islands consists of extremely fine twins^18^. Moreover, the island is harder than the ferrite matrix, and facilitating debonding, cracking, and crack initiation, can lead to a decrease in the toughness of the steel^19^.

### Effect of microstructure on mechanical properties

Table 3 shows the tensile properties of the SA508Gr.4 N steel in the quenched and tempered (QT) state and the after step-cooling heat treatment (QT-S.C) state. Two tests were carried out for each process. For both states, the measured ultimate tensile strength (UTS: ∼760 MPa) and yield strength (YS: ∼630 MPa) of the fast cooling (60 °C/min) sample were slightly higher than those (UTS: ∼740 MPa, YS: ∼600 MPa) of the slow cooling (4.4 °C/min) sample.

**Table 3:** Tensile properties of the SA508Gr.4N steel under different states.

State	QCR (°C/min)	Microstructure	Sample No.	UTS (MPa)	YS (MPa)	*A* (%)	*Z* (%)
QT	60	Martensite	QT-A-1	760	635	24	80
			QT-A-2	766	639	25	80
	4.4	Bainitie	QT-B-1	738	594	24	75
			QT-B-2	740	602	24	81
QT-S.C	60	Martensite	QT-S.C-A-1	751	625	22	79
			QT-S.C-A-2	759	631	26	81
	4.4	Bainitie	QT-S.C-B-1	725	590	25	80
			QT-S.C-B-2	729	594	25	76

After the step-cooling heat treatment, the strength of each sample decreases, but the strength of the fast cooling samples is still significantly higher (∼25 MPa) than that of the slow cooling samples. ASME specification for mechanical properties of SA508Gr.4N steel stipulates UTS and YS values of 725 MPa and 585 MPa, respectively. The test (i.e., SA508Gr.4N) steel meets the requirements of the ASME specification. Furthermore, the difference in the microstructures has no effect on the ductility, as evidenced by a total elongation (*A*) and a reduction in area (*Z*) of 24% and 78%, respectively, for both states. These values are higher than those stipulated in the ASME requirements (*A* ≥ 18%, *Z* ≥ 45%).

**Figure 4:**
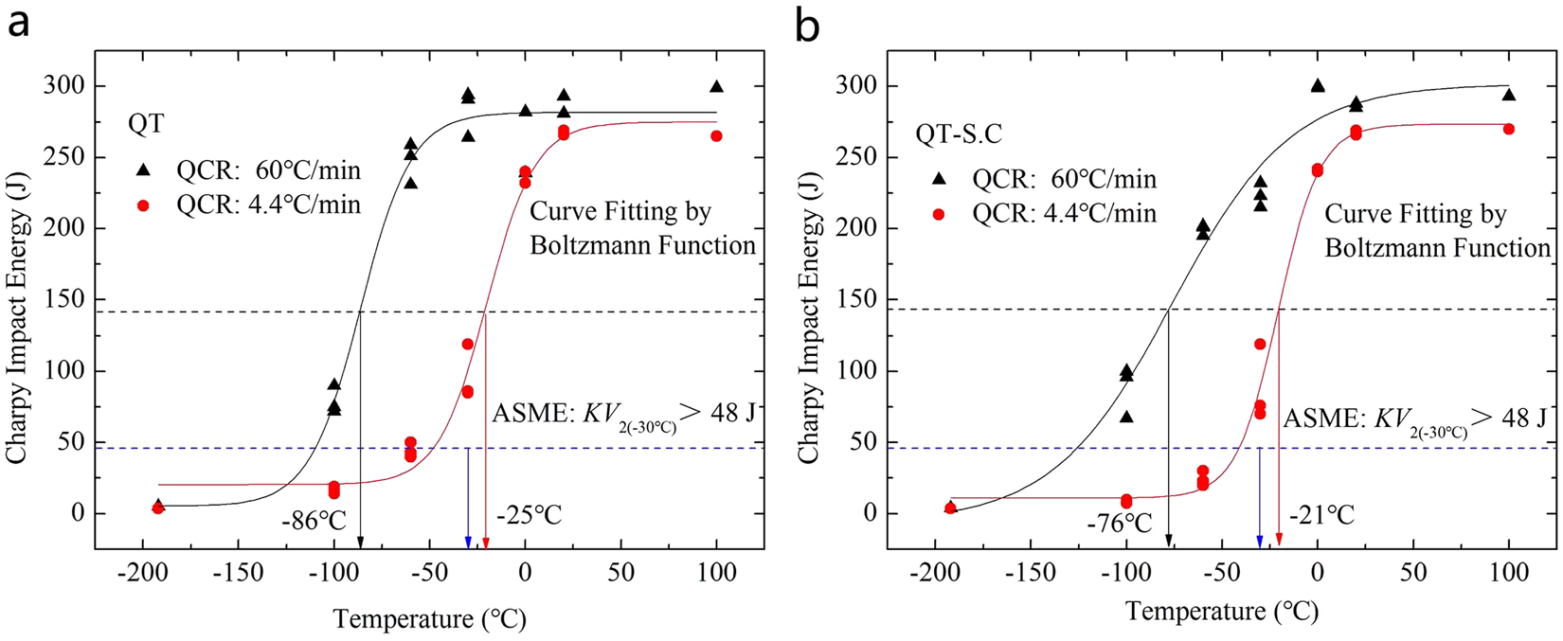
Charpy energy curves of the SA508Gr.4N steel under different states: (**a**) QT; (**b**) QT-S.C.

The temperature dependence of the Charpy impact energy of the SA508Gr.4N steel is shown in Fig. 4. The Charpy energy curves can be divided into three regions corresponding to the: upper shelf energy (USE), change in temperature, and lower shelf energy. The curves are basically S-shaped. When the test temperature is reduced, the impact force decreases and the impact energy decreases rapidly at the transition temperature. When the temperature is reduced to a certain extent, the rate of change of the impact force decreases gradually and becomes stable at a relatively low value. ASME specifies an impact performance of >48 J at a test temperature of −30 °C^17^. With the increase of QCR, the Charpy energy curves shifted to the lower-temperature zone on the left, indicating an increased toughness (Fig. 4). The impact energy at −30 °C of the QT and the QT-S.C states at a quenching cool rate of 4.4 °C/min were higher than 78 J and 70 J, respectively, above the values indicated by ASME.

The USE and ductile-to-brittle transition temperature (DBTT) of different states were determined by fitting the Boltzmann function fitting (Table 4). As the table shows, irrespective of the heat-treatment state, the USE increases gradually and the DBTT decreases when the QCR is increased. This indicates that the impact toughness of SA508Gr.4N steel can be improved by increasing the QCR. After the step-cooling heat treatment, the USE and DBTT both increase, and ΔDBTT increases with increasing QCR. This indicates that martensitic microstructures are more susceptible to temper embrittlement than bainitic microstructures^12,13^.

**Table 4:** Impact properties associated with the QT and QT-S.C states of the of SA508Gr.4N steel.

State	QCR (°C/min)	Microstructure	USE (J)	DBTT (°C)	△DBTT (°C)
QT	60	Martensite	280	−86	—
	4.4	Bainitie	258	−25	—
QT-S.C	60	Martensite	301	−76	10
	4.4	Bainitie	273	−21	4

### Microstructure of the SA508Gr.4N steel during tempering

**Figure 5:**
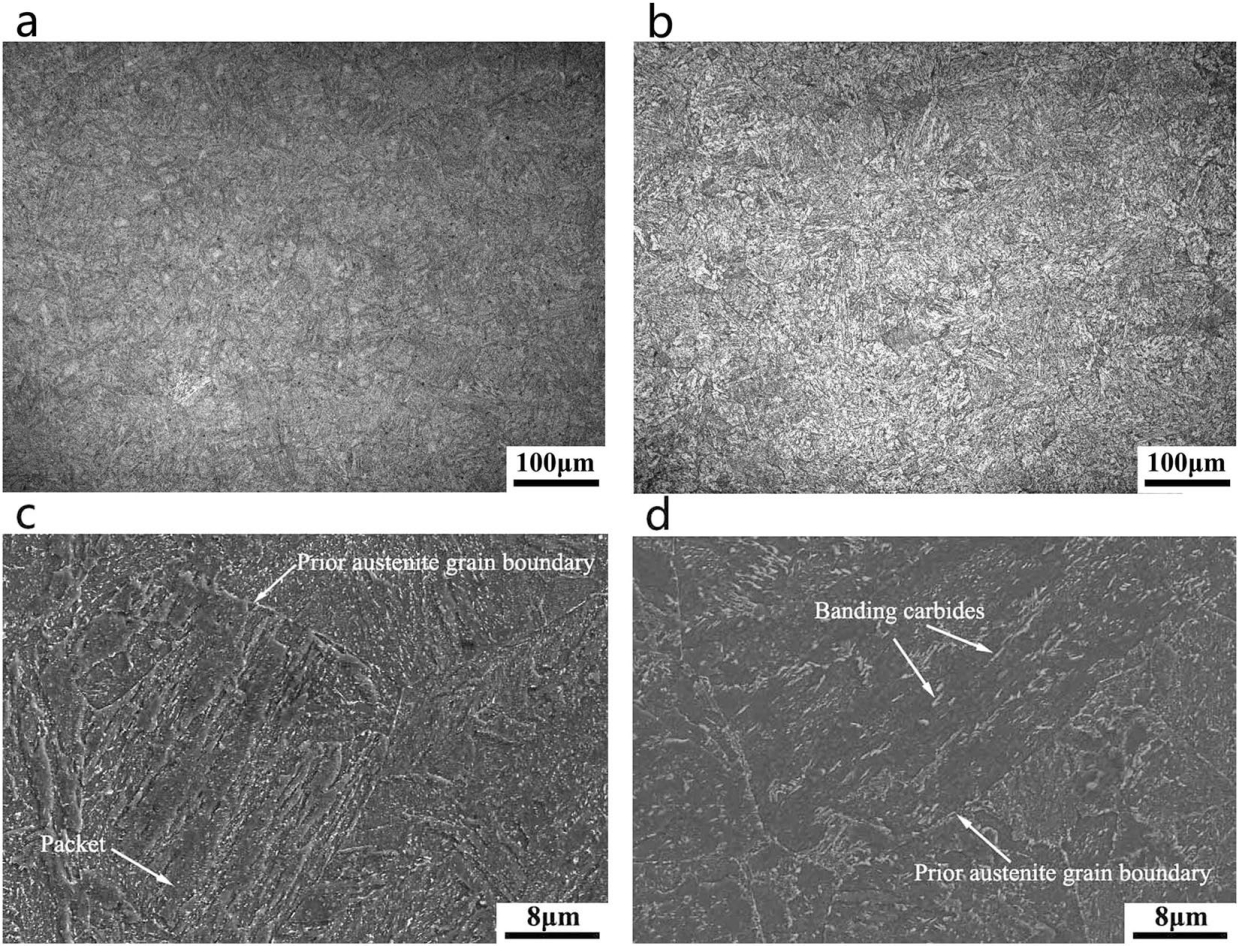
Optical and SEM micrographs of the SA508Gr.4N steel after tempered:(**a**,**c**) Martensite; (**b**,**d**) Bainite.

**Figure 6:**
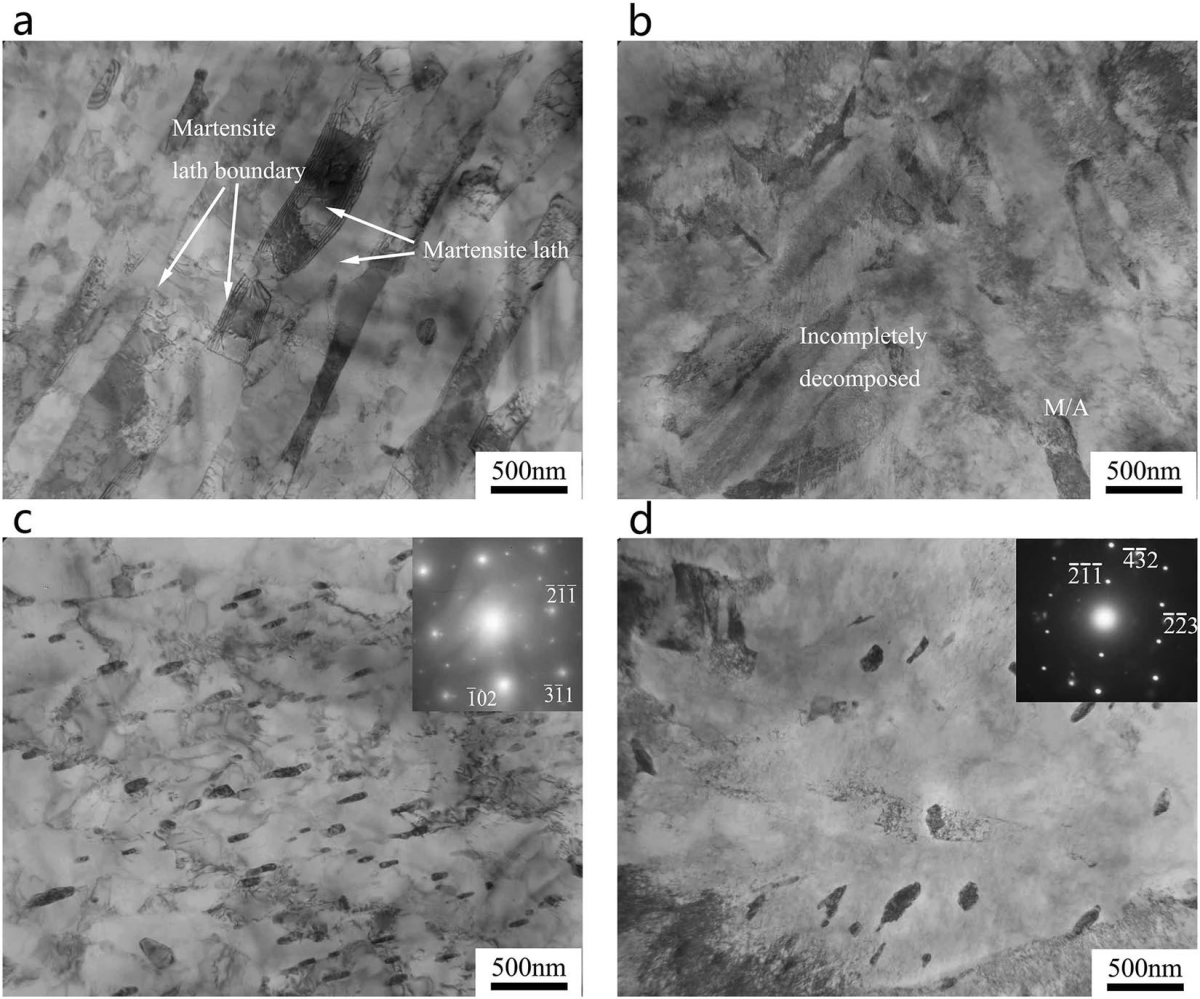
TEM micrographs of the SA508Gr.4N steel after tempering: (**a**,**c**) Martensite; (**b**,**d**) Bainite.

The microstructure of the SA508Gr.4N steel tempered at 620 °C for 8 h is shown in Fig. 5 and Fig. 6. As Fig. 5 shows, the tempered microstructure consists mainly of carbides. The carbides in tempered martensite are fine and evenly dispersed, whereas those in the tempered bainite are needle-like and clustered.

After tempering, martensite lath widths of 80 nm–450 nm, and lath widths increases slightly, as shown in Fig. 6(a). A large fraction of the M/A decomposes into ferrite and carbide, but a small fraction of incompletely decomposed M/A islands persists (see Fig. 6b). The carbides in martensite are relatively small and dispersed in a short rod (Fig. 6c), whereas the carbides in bainite are relatively coarse (Fig. 6d). The diffraction spot used to determined the carbide content in both martensite and bainite allowed the identification of M_23_C_6_ carbides. Changing the cooling rate during quenching has no effect on the type of carbide formed, but only on its morphology. Previous studies have reported a linear dependence of the carbide size on the critical distance between a crack tip and a cleavage initiation site. Therefore, the coarse carbides are detrimental to the impact toughness of the material^20,21^.

### Microstructure of the SA508Gr.4N steel during the step-cooling heat treatment

**Figure 7:**
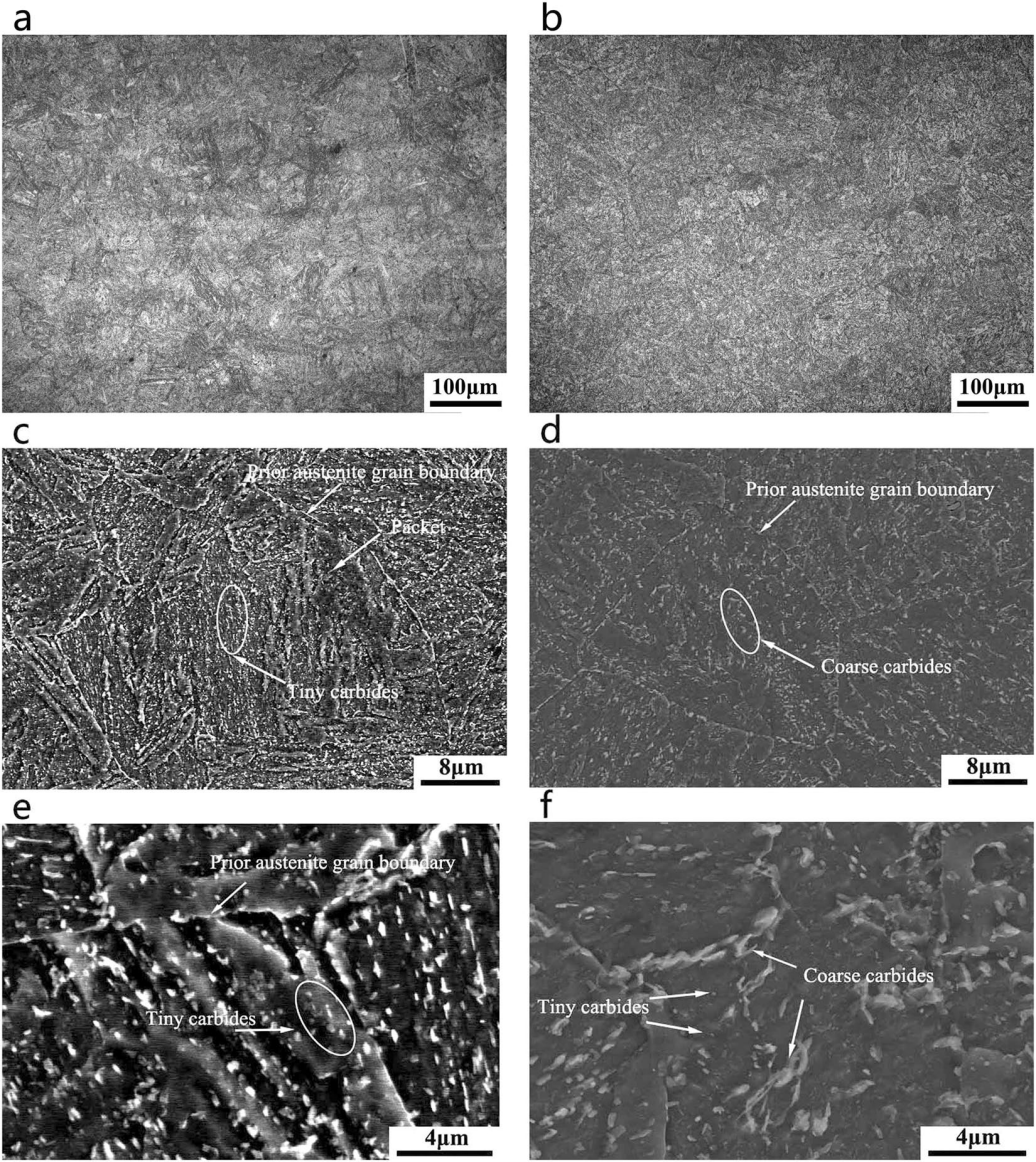
Optical and SEM micrographs of the SA508Gr.4N steel after step-cooling heat treatment: (**a**,**c**,**e**) Martensite; (**b**,**d**,**f**) Bainite.

**Figure 8:**
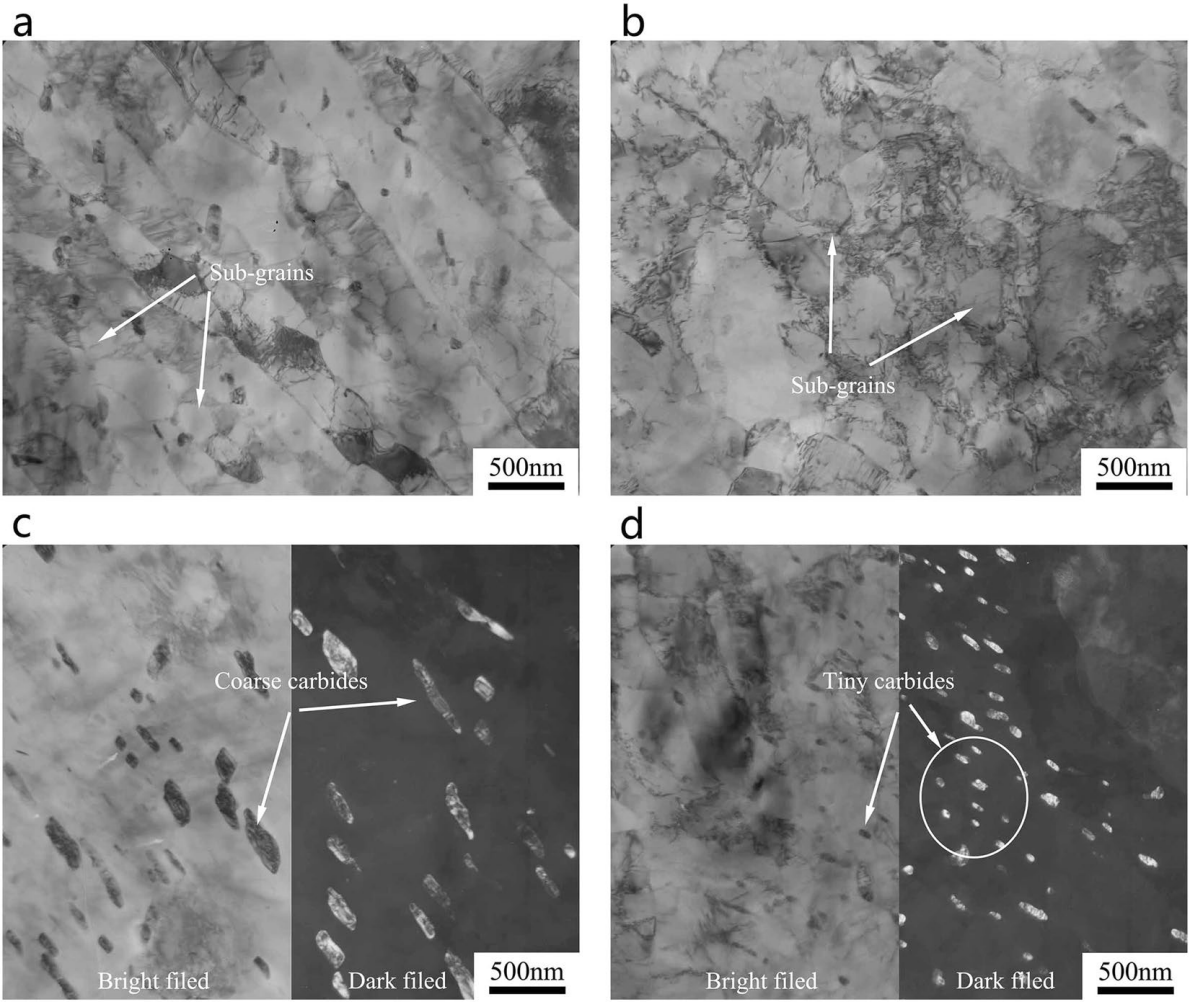
TEM micrographs of the SA508Gr.4N steel after step-cooling heat treatment: (**a**,**c**) Martensite; (**b**,**d**) Bainite.

The microstructure of the SA508Gr.4N steel after the step-cooling heat treatment is shown in Figs 7 and 8. As Fig. 7 shows, the carbides in the martensite are diffuse and small (Fig. 7c), and those in the bainite are discontinuous and disperse (Fig. 7d). The carbides in martensite are fine and uniformly distributed (Fig. 7e); on the other hand, the carbides distribution in bainite is not uniform and larger carbides were present (Fig. 7f). The distribution of carbides on the grain boundaries causes their embrittlement, promoting the fracture of the material along the grain boundaries. The grain boundary carbides in bainite are coarser than those in martensite, and larger carbides will reduce the critical fracture stress and decrease the toughness of the material.

After prolonged thermal insulation, clear martensite laths persist in the microstructure and a small fraction of sub-grains (Fig. 8a) is formed. Many sub-grains are formed in the bainite (Fig. 8b). Large carbides are distributed in the martensite lath boundary (Fig. 8c) and small carbons are dispersed in the bainite ferrite matrix (Fig. 8d). The carbides are distributed either on the martensite lath or the bainite ferrite matrix, and will maintain a certain coherent relationship with the matrix. The stress field produced by the precipitates can change the stress distribution at the crack tip and prevent the nucleation and propagation of cracks. However, if the carbide itself is very small and its strength is not high, a dislocation motion occurs and the dislocation concentrated in the carbide forms a crack, which will reduce the toughness of the steel. If the dislocation cannot cut through the carbide, the Orowan bypassing mechanism will not significantly reduce the toughness of the material.

## Discussion

### Effect of the microstructure on the impact toughness

The experimental results reveal that, for both the QT and QT-S.C states, the impact properties of the martensitic microstructures are improved more significantly than those of the bainitic microstructures.

The impact toughness of a low alloy steel is (in general) strongly dependent on crack initiation. In addition, the effective grain size and HAGBs distribution in the steel have a significant effect on the crack propagation path^22^. Morito *et al*.^23^ suggested that the block size plays a key role in the strength and toughness of low carbon steels.

**Figure 9:**
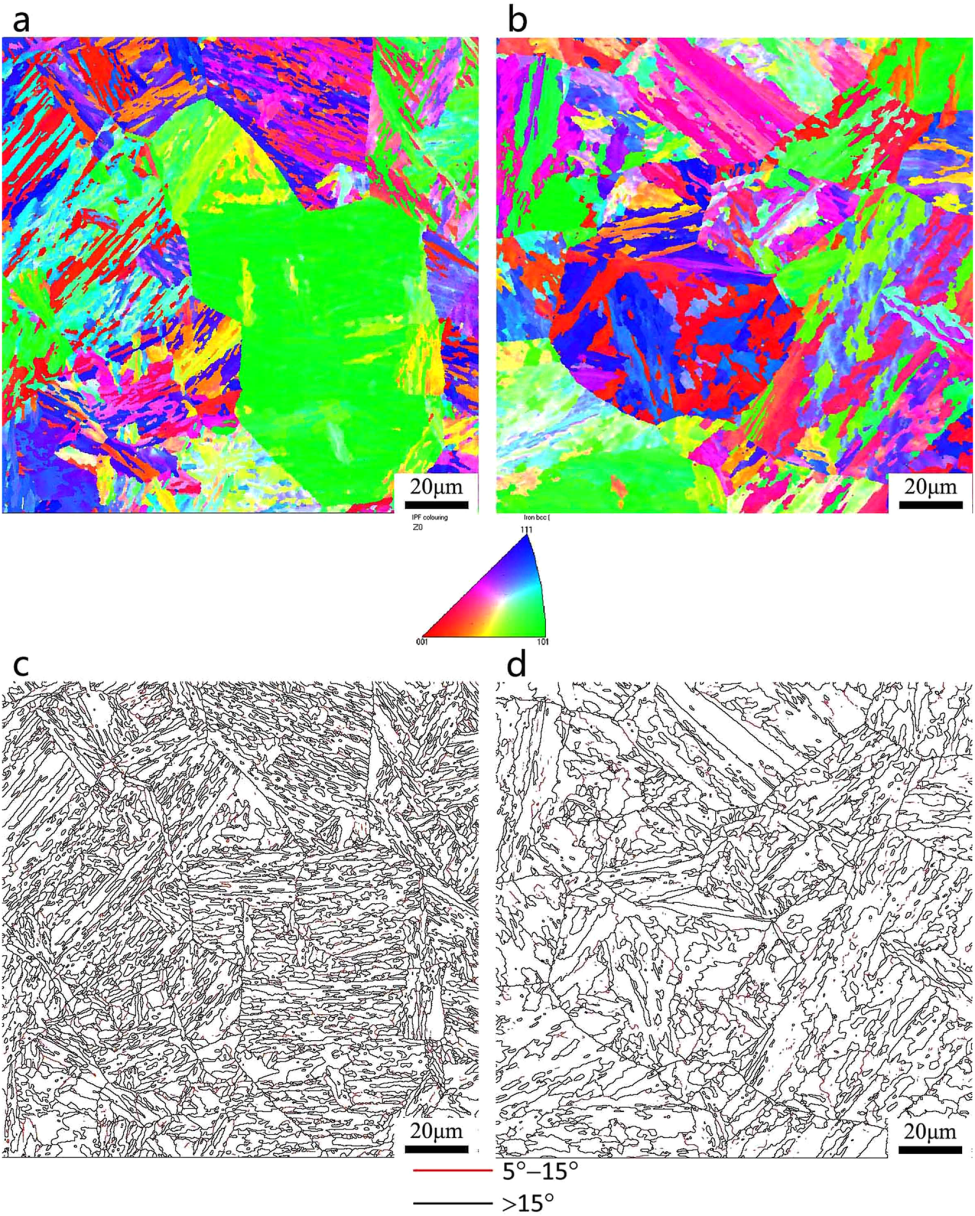
Orientation image maps and grain boundary maps of SA508Gr.4N steel after tempering: (**a**,**c**) Martensite; (**b**,**d**) Bainite.

**Figure 10:**
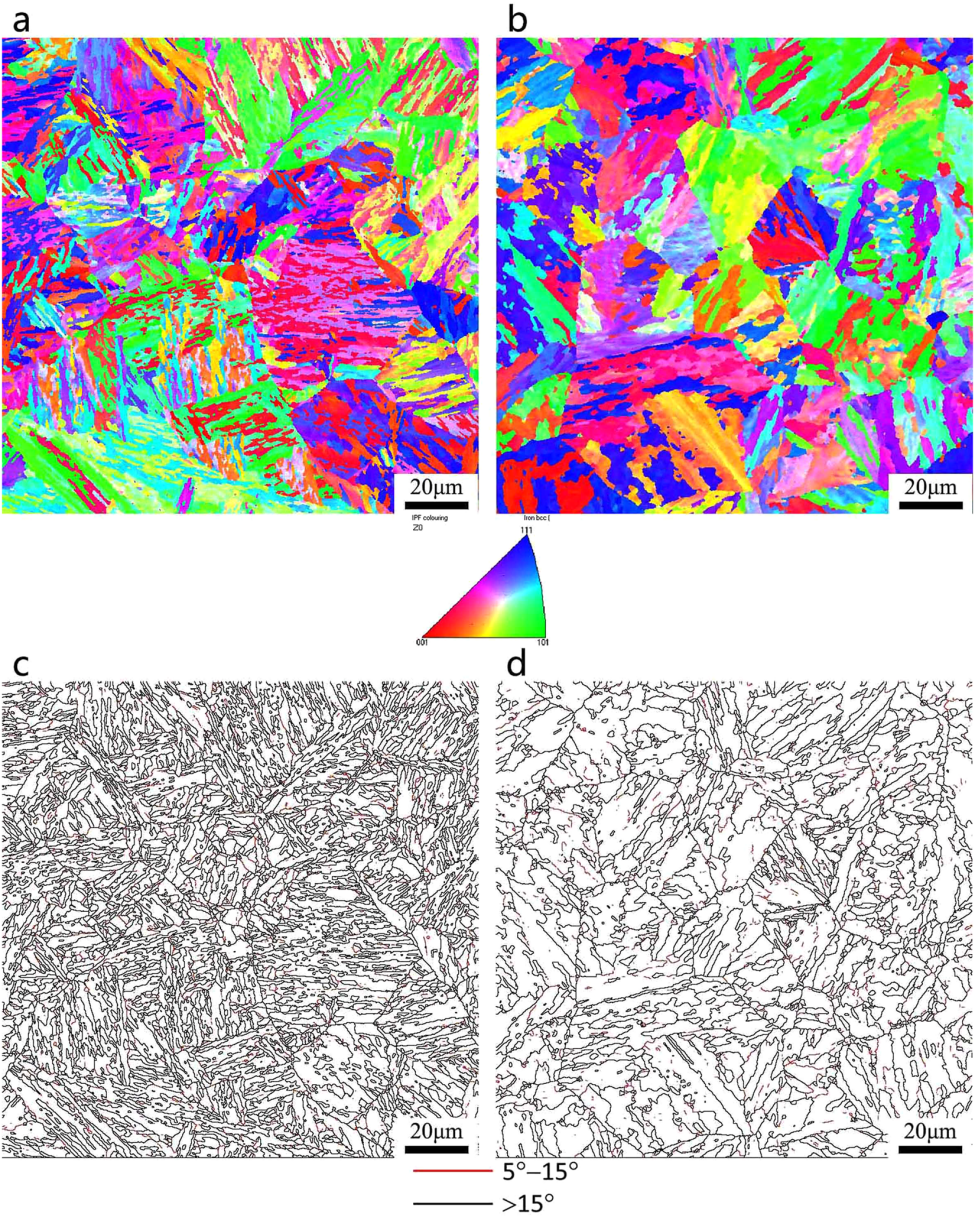
Orientation image maps and grain boundary maps of SA508Gr.4N steel after step-cooling heat treatment: (**a**,**c**) Martensite; (**b**,**d**) Bainite.

The EBSD orientation maps and grain boundary maps of the SA508Gr.4N steel are shown in Fig. 9 (QT) and Fig. 10 (QT-S.C). The samples in the QT state consist of ∼2,600 martensitic blocks of 0.2–14.4 μm. Approximately 1400 bainitic blocks (size: 0.2–31.4 μm) were considered. The samples in the QT-S.C state consist of ∼2400 martensitic blocks of 0.2–12 μm. Approximately 1200 bainitic blocks (size: 0.2–30.4 μm) were considered. The block width and the fraction of HAGBs, determined via statistical analyses, are shown in Table 5.

**Table 5:** The block width and high angle grain boundary of SA508Gr.4N Steel under different state.

State	QCR (°C/min)	Microstructure	Block width (μm)	High-angle grain boundaries (>15°) (%)
QT	60	Martensite	1.685	41.56
	4.4	Bainitie	2.887	27.04
QT-S.C	60	Martensite	1.789	37.21
	4.4	Bainitie	3.034	24.31

After tempering, the average block width increases slightly from 1.306 μm to 1.744 μm when the QCR decrease from 60 °C/min to 4.4 °C/min. After the step-cooling heat treatment (Fig. 10(a,b)), the block width of the martensite is still smaller than that of the bainite. Pickering *et al*.^24^ have proposed the following relation for the DBTT:

1 $$DBTT=A-K\cdot \,\mathrm{ln}\,{d}^{-1/2}$$

where *A* and *K* are constants, and *d* is the effective size. The block width and the DBTT of the SA508Gr.4 N steel can be refined and significantly reduced, respectively, by increasing the QCR.

Grain boundary maps of the SA508Gr.4N steel are shown in Figs 9(c,d) and 10(c,d). Low angle grain boundaries (LAGBs) characterized by misorientations of 5°–15°, and HAGBs characterized by misorientations of >15° are shown as red lines and black lines, respectively.

According to previous studies^25,26^, the HAGBs determine the effective grain size, as they play a vital role in blocking crack propagation. The grain boundary energy of the HAGBs is much higher than that of the LAGBs, because in the former a higher number of atoms deviates from its equilibrium position. When the cracks propagate towards the HAGBs, their direction changes multiple times due to the irregular arrangement of the atoms, and the crack propagation energy is consumed. Therefore, HAGBs can effectively hinder the propagation of cracks, reduce the crack growth rate, improve the fracture toughness of the steel, and improve the impact energy. This will result in the Charpy energy curves moving towards the low temperature zone (Fig. 4), reducing the DBTT. HAGBs can disrupt crack propagation, i.e., the propagation direction changes at these boundaries and the crack propagation path increases. As such, the toughness of the steel increases with increasing fraction of HAGBs. Furthermore, according to Hwang *et al*.^27^. HAGBs serve as effective obstacles to cleavage-crack propagation, thereby resulting in a decrease in the DBTT.

For both the QC and QC-S.C states, the fraction of HAGBs in the martensitic microstructures is ∼10% higher than that in the bainitic microstructures. The block width and the fraction of HAGBs can be refined and increased, respectively, by increasing the QCR. Therefore, the fraction of HAGBs increases with decreasing block width and the total impact toughness of the SA508Gr.4N steel is thereby maximized.

### Effect of the microstructure on the temper embrittlement

**Figure 11:**
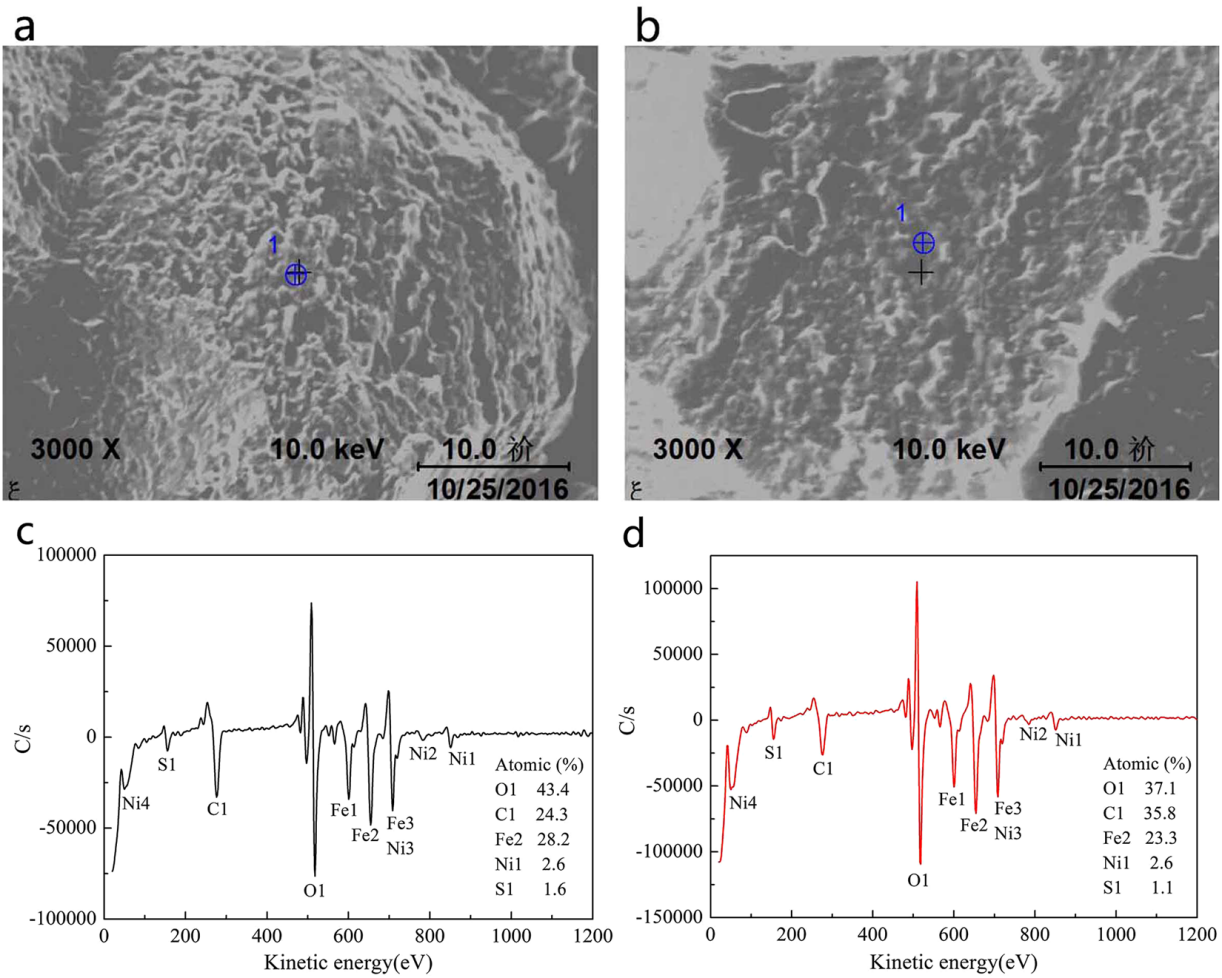
Auger electron energy spectra showing grain boundary segregation of elements after QT-S.C: (**a**,**c**) Martensite; (**b**,**d**) Bainite.

Temper embrittlement is usually attributed to grain boundary segregation of impurity elements^28,29^. The elemental concentration at the grain boundaries in samples cooled at 60 °C/min (martensitic) and 4.4 °C/min (bainitic) after the step-cooling heat treatment was determined via AES of the fracture surface of two specimens. Figure 11 reveals the segregation of O, C, and S.

Despite the high Mn and Si content, these elements remained in inside the grain rather than becoming segregated at the boundaries. The Auger analysis technology has an atomic detection limit of a single atomic layer (atom percentage of 1/1000). Therefore, if the concentration of elements involved in surface segregation is higher than the detection limit there will be a signal. On the contrary, if the concentration is below the detection limit, no information is obtained from the analysis. The average boundary levels of S in martensite and bainite are ∼1.6 at% and 1.1 at% respectively. Barrett *et al*.^30^ reported that S leads to a significant increase in the temper embrittlement of low alloy steel. Furthermore, Harrelson *et al*.^31^ reported that S promotes the formation of non-metallic inclusions and intergranular fracture in Cr-Mo steel, and is more effective than P in inducing embrittlement. S segregation occurs preferentially in correspondence of the high-energy boundaries, such as the prior austenite grain boundaries. Therefore, differences between the segregation of S in tempered martensite and tempered bainite result mainly from the different portions of high-energy random boundaries at the packet boundaries. The level of S segregation in martensite is significantly higher than that in bainite and, hence, martensite will be more susceptible to temper embrittlement.

## Conclusions

The effect of microstructure on the impact toughness and temper embrittlement of SA508Gr.4N steel was investigated. The results are summarized as follows:

1. The strength of the martensitic structure is ∼25 MPa higher than that of bainitic structure. Similarly, the impact toughness of the martensitic structure is better than that of the bainitic structure, and the DBTT values differ by 60 °C.
2. Compared with the bainitic structure, the martensitic structure consists of finer blocks (i.e., block width is smaller) and more HAGBs. This leads to an improved crack-propagation path at break, and the propagation direction of the cleavage crack is deflected more frequently (than in the bainitic structure), resulting in a high impact toughness of the martensitic structure.
3. The segregation of S is significantly higher in the martensitic structure than in the bainitic structure and, hence, the martensitic structure will be more susceptible to temper embrittlement.
